# The Value of Alternatives Assessment

**DOI:** 10.1289/ehp.1611248

**Published:** 2016-03-01

**Authors:** Christopher P. Weis

**Affiliations:** National Institute of Environmental Health Sciences, National Institutes of Health, U.S. Department of Health and Human Services, Bethesda, Maryland, USA

The pages of *EHP* are replete with studies documenting the potential human health and ecosystem impacts of toxic chemicals. Such research is critical to understanding chemical risks and developing prevention strategies to reduce those risks. Intramural research at the National Institute of Environmental Health Sciences (NIEHS) as well as externally funded studies have led to a better understanding of mechanisms by which toxic chemicals may cause illness along with advanced tools to more rapidly assess risks. As knowledge of the potential impacts of specific chemicals increases, so do market and regulatory pressures to replace those chemicals with safer alternatives.

Regrettable substitutions, implemented without adequate screening, can too quickly enter the market if we neglect to develop processes to evaluate those alternatives. For example, research at the NIEHS has shown that many of the substitutes for certain brominated flame retardants may be as concerning as the chemicals they are replacing (Jarema et al. 2015). It is critical, therefore, that we establish thoughtful yet efficient processes to guide the transition to safer chemicals and products. This is the emerging field of alternatives assessment, which focuses on identifying, comparing, and selecting safer alternatives to chemicals of concern on the basis of their hazards, performance, and economic viability. By focusing on function, both chemical and nonchemical options may be considered to achieve the desired property, such as fire retardancy, stain resistance, or degreasing ability.

In this issue of *EHP*, Jacobs et al. (2016) provide a detailed analysis of 20 alternatives assessment frameworks, many developed by governments and industry to guide the selection of safer alternatives. Included in their analysis is the recently published National Research Council (NRC) *Framework to Guide Selection of Chemical Alternatives* (2014), which, much like the NRC’s famous “Red Book” on risk assessment (1983), can serve as a guide and foundation for this emerging science policy field.

In March 2015 the NIEHS, along with the U.S. Environmental Protection Agency, hosted the International Symposium on Alternatives Assessment: Advancing Science and Practice (http://www.saferalternatives.org/). That workshop brought together more than 100 government, academic, industry, and nonprofit scientists from the United States, Canada, and Europe to explore ways to build a more coordinated community of practice around alternatives assessment. Participants discussed gaps in knowledge and methods, and developed the elements of a research agenda to support this growing field.

Identifying safer alternatives makes sense for environmental health and safety as well as for economics. At the National Institutes of Health, we are doing this internally through the Substances of Concern Reduction Initiative (http://orf.od.nih.gov/EnvironmentalProtection/Pages/NIH-Substances-of-Concern-Reduction-Initiative.aspx), established to support institute purchasing of safer substitutes for chemicals of concern used in our laboratories, clinical centers, maintenance, and buildings. The NIEHS is committed to using its scientific research to support the design, evaluation, and adoption of safer chemicals. The NRC Framework (2014) identified a number of ways in which high-throughput and *in silico* research on chemical hazards and potential exposures could significantly enhance the alternatives assessment process, filling in important data gaps. Such research can also help in applying 21st-century toxicology to the design of new green chemistry solutions that are more healthful for people and the environment.

Inherent in alternatives assessment is the idea that when we have reasonable evidence that a chemical could be problematic, thoughtful steps are taken to evaluate materials, processes, and technologies to identify a safer substitute. At the NIEHS we look forward to working with the scientific and regulatory communities to help advance this important field.
